# *E. urophylla × E. grandis* high-quality genome and comparative genomics provide insights on evolution and diversification of eucalyptus

**DOI:** 10.1186/s12864-023-09318-0

**Published:** 2023-04-28

**Authors:** Chao Shen, Limei Li, Lejun Ouyang, Min Su, Kexin Guo

**Affiliations:** College of Biological and Food Engineering, Guangdong University of Petrochemical Technology, Maoming, China

**Keywords:** *E. urophylla × E. grandis*, Genome sequences, Comparative genomics, Eucalyptus

## Abstract

**Background:**

*Eucalyptus urophylla × Eucalyptus grandis*, an economically important forest tree, provides important raw material for energy and reduces damage to native forests. However, the absence of a high-quality *E. urophylla × E. grandis* reference genome has significantly hindered its evolution and genetic analysis.

**Results:**

We successfully presented a high-quality reference genome of *E. urophylla × E. grandis* (545.75 Mb; scaffold N50, 51.62 Mb) using a combination of the Illumina, PacBio HiFi, and Hi-C sequencing platforms. A total of 34,502 genes and 58.56% of the repetitive sequences in this genome were annotated. Using genome evolution analyses, we identified a recent whole-genome duplication (WGD) event in *E. urophylla × E. grandis*. We further found that gene families associated with starch and sucrose metabolism, flavonoid biosynthesis, and plant–pathogen interaction were significantly expanded in *E. urophylla × E. grandis*. Moreover, comparative genomic and evolutionary analyses showed large structural variations among the different chromosomes of the 34 Eucalyptus accessions, which were divided into six clades.

**Conclusions:**

Overall, our findings provide a valuable resource for expanding our understanding of the *E. urophylla × E. grandis* genome evolution, genetic improvement, and its comparative biology.

**Supplementary Information:**

The online version contains supplementary material available at 10.1186/s12864-023-09318-0.

## Background

Eucalyptus, belonging to the Myrtaceae family, grows rapidly, is widely cultivated worldwide (over 20 million hm^2^) [1], and has a high economic value because of its wide range of uses. Introduced to China more than 120 years ago, eucalyptus provides an annual wood output of more than 40 million m^3^ and more than 50% of China’s pulping materials [2].

*Eucalyptus urophylla* is a hybrid species with rapid growth and strong stress resistance among eucalyptus trees. It is the dominant species in artificial forestation in China and occupies the largest area.

*E. urophylla × E. grandis* is a successful hybrid species of Eucalyptus and is the leading variety of artificial afforestation, occupying the largest area in China with rapid growth and strong stress resistance. The transfer of these excellent economic and agronomic traits to other Eucalyptus species via interspecific hybridization is important for broadening the genetic diversity of eucalyptus. However, recent research on *E. urophylla × E. grandis* has focused on wood processing, with few studies exploring the genomic biology of these superior qualities. To compensate for this deficiency and overcome these obstacles in basic and applied biology research, it seems essential to assemble a high-quality reference genome of *E. urophylla × E. grandis*.

Comparative genomics provides important information on evolutionary biology and useful information on interspecific genomic differences [3–5]. For example, identifying conserved genome structures, inferring common ancestry, and analyzing genomic similarities and differences are important for evolutionary genetics and the transmission of genetic information [6]. The increasing number of genomic resources allows us to use comparative genomics to gain new insights into the evolutionary variation of individual genes and gene families [7, 8] in whole genomes [9–11]. Eucalyptus belongs to the family Myrtaceae and includes more than 700 different species [12, 13], most of which belong to the Eucalyptus subgenus [14, 15]. Genomic research on eucalyptus is relatively underdeveloped. The reference genome of *E. grandis* was published only in 2014 [16], which has greatly hindered genomics and population genetics research on eucalyptus. Therefore, little research has been conducted on eucalyptus in this area.

Here, we reveal the high-quality reference genome of *E. urophylla × E. grandis* using a combination of Illumina sequencing, PacBio HiFi sequencing, and Hi-C sequencing platforms with a size of 545.75 Mb, containing 34,502 protein-coding genes. Repetitive elements occupied 58.56% of the genome. Comparative genomic analyses revealed that *E. urophylla × E. grandis* had recently undergone a WGD event and large-scale structural variation among the 34 Eucalyptus accessions. The results presented in this study provide a foundation for *E. urophylla × E. grandis* genomic studies seeking to affirm the genetic variation, genome evolution, and genealogical structure of Eucalyptus as well as for breeding studies for the genetic improvement of *E. urophylla × E. grandis*.

## Results

### The high-quality *E. urophylla × E. grandis* genome

**Fig. 1:**
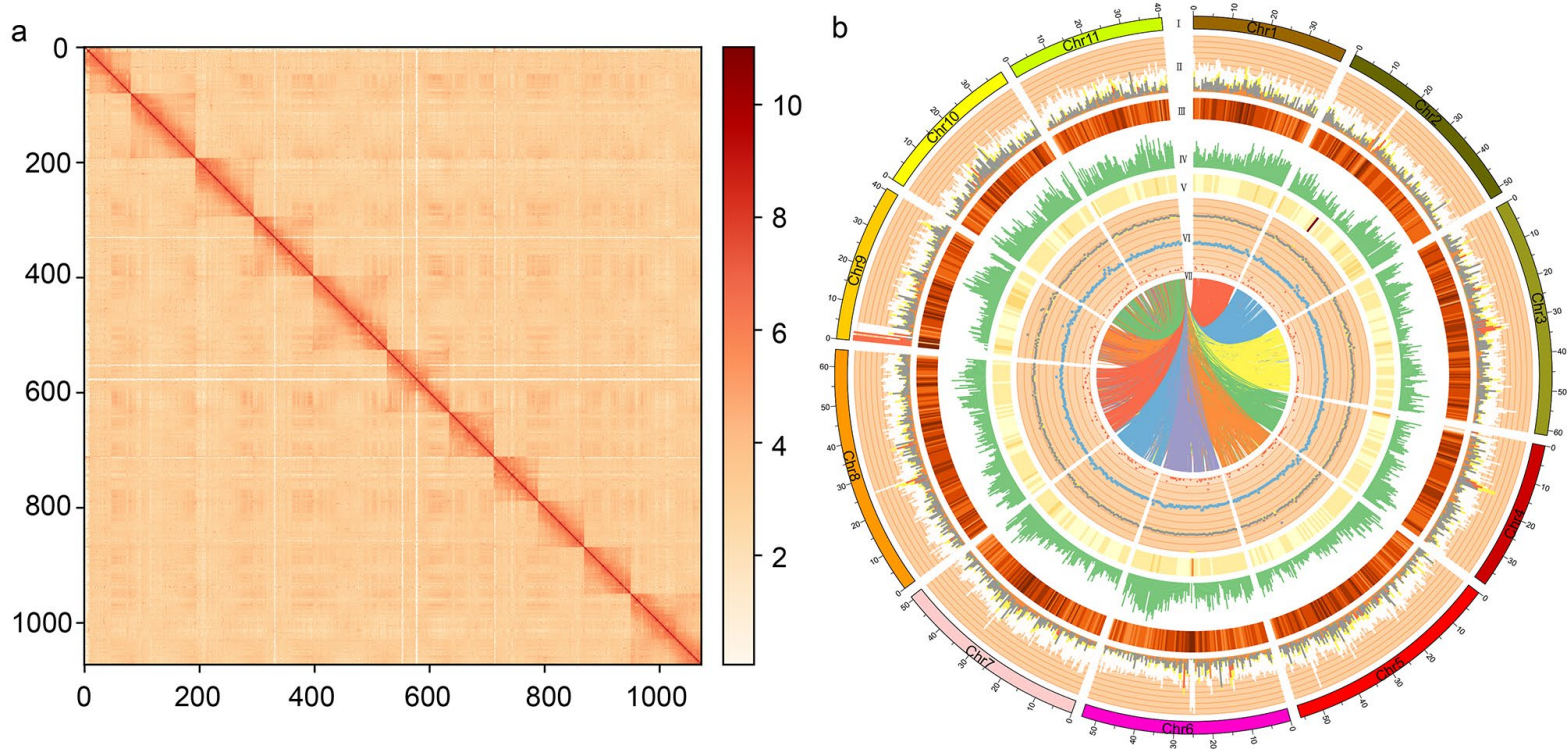
The global landscape of *E. urophylla × E. grandis*. (**a**) Hi-C interaction heatmap of *E. urophylla × E. grandis*. (**b**) Genomic landscapes of *E. urophylla x E. grandis*. (I–VII are chromosomes, different types of TE, TE heatmap (500 kb), gene density (500 kb), GC content heatmap (500 kb), different types of noncoding RNAs, and gene pairs of *E. urophylla × E. grandis*

We obtained 78.13 gigabases (Gb ~ 142.31-fold) Illumina paired-end sequences (Supplementary Table 1), which indicated that the estimated *E. urophylla × E. grandis* genome size was 549 Mb, with 2.16% heterozygosity, using K-mer analysis (Supplementary Table 2). We sequenced *E. urophylla × E. grandis* by generating 28.16 gigabases (Gb) PacBio high-fidelity (HiFi) data, yielding a preliminary genome assembly of 545.93 Mb (contig N50, 39.94 Mb) with a GC content of 39.89% (Table 1 and Supplementary Table 1). To obtain a high-quality genome of *E. urophylla × E. grandis*, a total of 59.32 Gb of data were obtained with 108.69-fold genome coverage by Hi-C sequencing, which was used to construct a chromosome interaction heatmap. The total number of scaffolds was 209, the longest being 68 Mb. Subsequently, the final assembly captured a 545.75 Mb genome sequence (scaffold N50, 51.62 Mb) containing 34,502 protein-coding genes with 58.56% of repeat sequences. According to the Hi-C contact maps, 98.29% of the entire genome was organized and divided into 11 chromosomes (Fig. 1a; Table 1). The longest chromosome was 64.4 Mb (Chr8) and the shortest was 38.5 Mb (Chr4) (Supplementary Table 3). The mean long terminal repeat (LTR) assembly index (LAI) score was 18.51. The genetic region assembly integrity of the highly conserved core proteins was supported by 98.4% (1588) (Table 1 and Supplementary Table 4) using the Benchmarking Universal Single-Copy Orthologs (BUSCO) analysis. This further confirms the integrity and high quality of the genome assembly.

**Table 1:** Summary of *Eucalyptus urophylla × E. grandis* genome assembly

Genomic feature	*E. urophylla* × *E. grandis*
PacBio reads (Gb)	28.16
Hi-C reads (Gb)	59.32
Length of assemblies (Mb)	545,75
Longest scaffold (Mb)	62,03
Scaffolds number	209
Scaffold N50, Mb	51.62
Repeat sequence	58.56%
Complete BUSCOs	98.40%
Raw_LAI	12.88
LAI	18.51
GC content	39.89%
Number of genes	34,502

The genomic features of *E. urophylla × E. grandis* are shown in Fig. 1. Based on the highly contiguous *E. urophylla × E. grandis* genome, 34,502 protein-coding genes were identified, which was lesser than the number in *E. grandis* (36,349). The average gene and coding sequence (CDS) lengths were 5,148 bp and 1,218 bp, respectively. The average exon and intron lengths were 306.77 and 813.98 bp, respectively (Supplementary Fig. 1). Among the 34,502 predicted genes, 31,241 (90.55%) were functionally annotated from 10 known databases: InterPro (23,485 genes, 68.07%), GO (Gene Ontology; 16,385 genes, 47.49%), KEGG_ALL (30,405 genes, 88.13%), KEGG_KO (11,116 genes, 32.22%), Swiss-Prot (20,220 genes, 58.61%), TrEMBL (30,391 genes, 88.08%), TF (1,840 genes, 5.33%), Pfam (22,760 genes, 65.97%), NR (30,970 genes, 89.76%), and KOG (23,839 genes, 69.09%) (Supplementary Table 5). Among the predicted repetitive elements, 39.4% were long terminal repeats (LTRs), 14.00% were DNA transposons, and 5.98% were long interspersed nuclear elements (LINEs) (Supplementary Tables 7 and Supplementary Fig. 2). We identified 828 miRNAs, 9,657 rRNAs, 411 snRNAs, and 450 tRNAs (Supplementary Tables 8, Fig. 1bVI). The GC content was unevenly distributed (Fig. 1bV).

### Evolutionary analysis of the *E. urophylla × E. grandis* genome

**Fig. 2:**
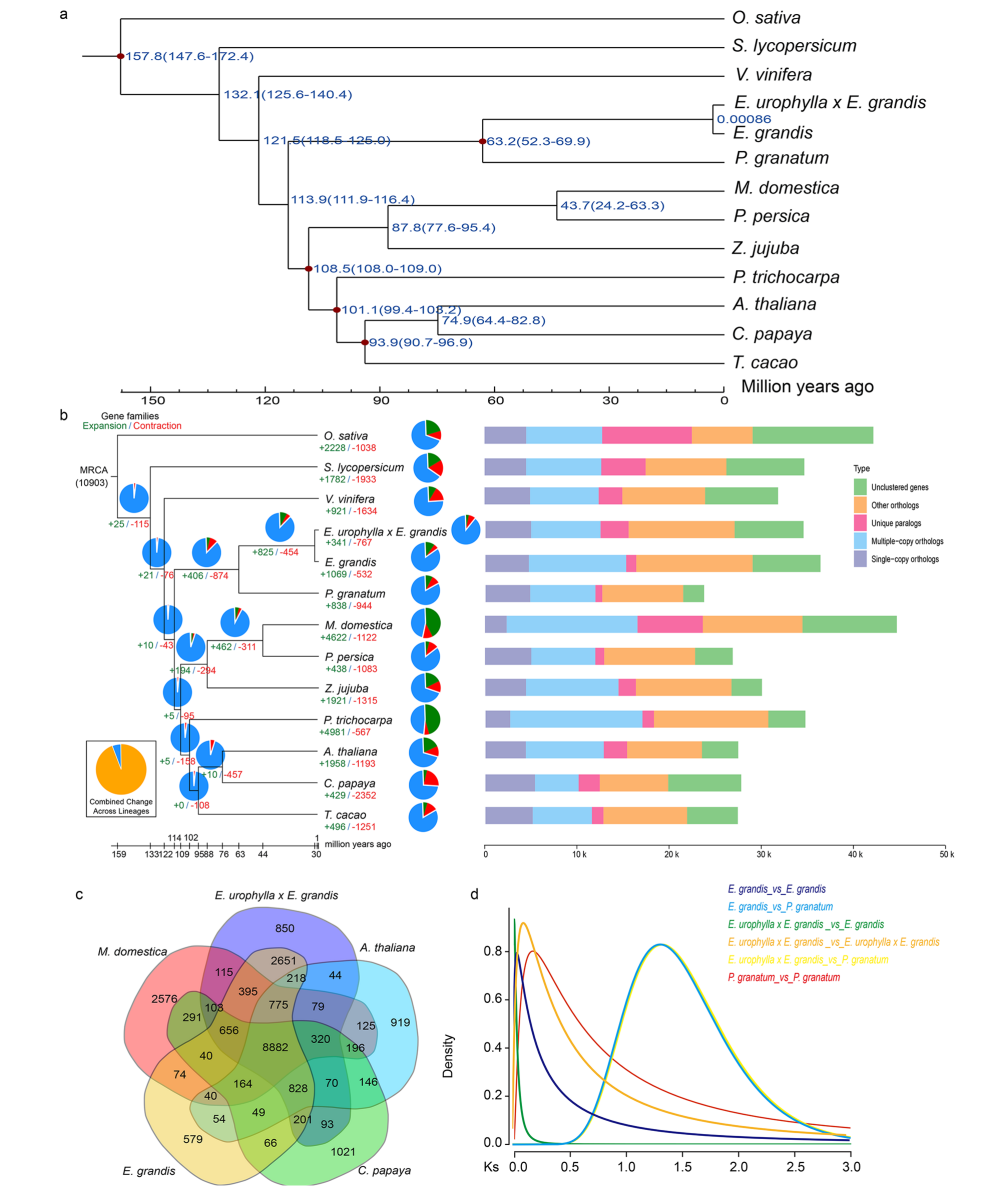
The *E. urophylla × E. grandis* genome evolution. (**a**) Phylogenetic tree construction and divergence time estimation for *E. urophylla × E. grandis* and 12 representative plants. (**b**) The gene family expansion (green), contraction (red), and gene copy number distribution. (**c**) Venn diagram showing the gene family clusters in *E. urophylla × E. grandis*, *M. domestica*, *A. thaliana*, *E. grandis*, and *C. papaya*. (**d**) Ks distribution in *E. grandis*, *P. granatum*, and *E. urophylla × E. grandis*

To investigate the *E. urophylla × E. grandis* genome evolution, we collected 12 other representative plant species genomes using comparative genomic analyses and identified the gene families, which revealed that there were 16,280 gene families and 3050 species-specific single-copy genes (Supplement Table 9). In addition, clustering analyses revealed that there were 27,032 genes in the families, accounting for 78.3% of the predicted genes in *E. urophylla × E. grandis*, which was similar to the % of predicted genes in *E. grandis* (Supplementary Table 9). Based on gene family analyses, we constructed a phylogenetic tree with 652 shared single-copy orthologs of the 13 species, which indicated that *E. urophylla × E. grandis* was most closely related to *E. grandis*, comprising a monophyletic group and specialized approximately 0.00086 million years ago (Mya), whereas the divergence time estimated between *Eucalyptus* and *P. granatum* was approximately 63.2 Mya (Fig. 2a).

Comparative evolutionary analysis of the gene families in 13 plant species showed that 341 gene families of *E. urophylla × E. grandis* showed significant expansion relative to the gene families of the most recent common ancestor, whereas 767 showed significant contraction (p < 0.01, Fig. 2b). We found that *E. urophylla × E. grandis* had fewer gene family expansions and more gene family contractions than the other Myrtaceae species (Fig. 2b), which is consistent with the lower gene number. We identified 4958 single-copy genes in *E. urophylla × E. grandis* via clustering analyses, accounting for 14.37%, which was similar to that in *E. grandis* (Fig. 2b). Gene ontology (GO) and Kyoto Encyclopedia of Genes and Genomes (KEGG) showed that the expanded gene families were involved in ion binding, plant–pathogen interactions, carbohydrate derivative binding, flavonoid biosynthesis, starch and sucrose metabolism, and pentose and glucuronate interconversion (Supplementary Figs. 3 and 4). However, functional analysis of the contracted gene families revealed that they were involved in the Toll-like receptor signaling pathway, NF-kappa B signaling pathway, cell recognition, protein modification process, ion binding, MAPK signaling pathway, and phenylpropanoid biosynthesis (Supplementary Figs. 5 and 6). The results of gene family clustering showed that *E. urophylla × E. grandis* and *E. grandis* shared more gene families than *M. domestica*, *A. thaliana*, and *C. papaya*, which was consistent with their phylogenetic relationships (Fig. 2b, c).

To estimate WGD events, *E. grandis* and *P. granatum* were selected and their synonymous nucleotide substitutions (Ks) were characterized. In addition, we found that a WGD event recently occurred in *E. urophylla × E. grandis* genome after its divergence from *P. granatum* with the in-depth comparison genomic analyses (Fig. 2d). *E. grandis* and *P. granatum* also underwent genome-wide replication following divergence. Furthermore, based on the Ka/Ks ratios, we found that 113 candidate genes were under strict positive selection in *E. urophylla x E. grandis* (p < 0.05). GO and KEGG enrichment analysis showed that the positive selection genes were enriched in “DNA metabolic process,” “Protein-containing complex assembly,” “Cellular response to DNA damage stimulus,” “Fanconi anemia pathway,” “DNA replication,” “Cholesterol metabolism,” “Homologous recombination,” “Cell cycle,” and “Steroid biosynthesis,” indicating that they may improve DNA damage resistance and the related metabolic pathways in adverse environments (Supplementary Figs. 7 and 8).

### Comparative genomic analysis of *Eucalyptus* species

**Fig. 3:**
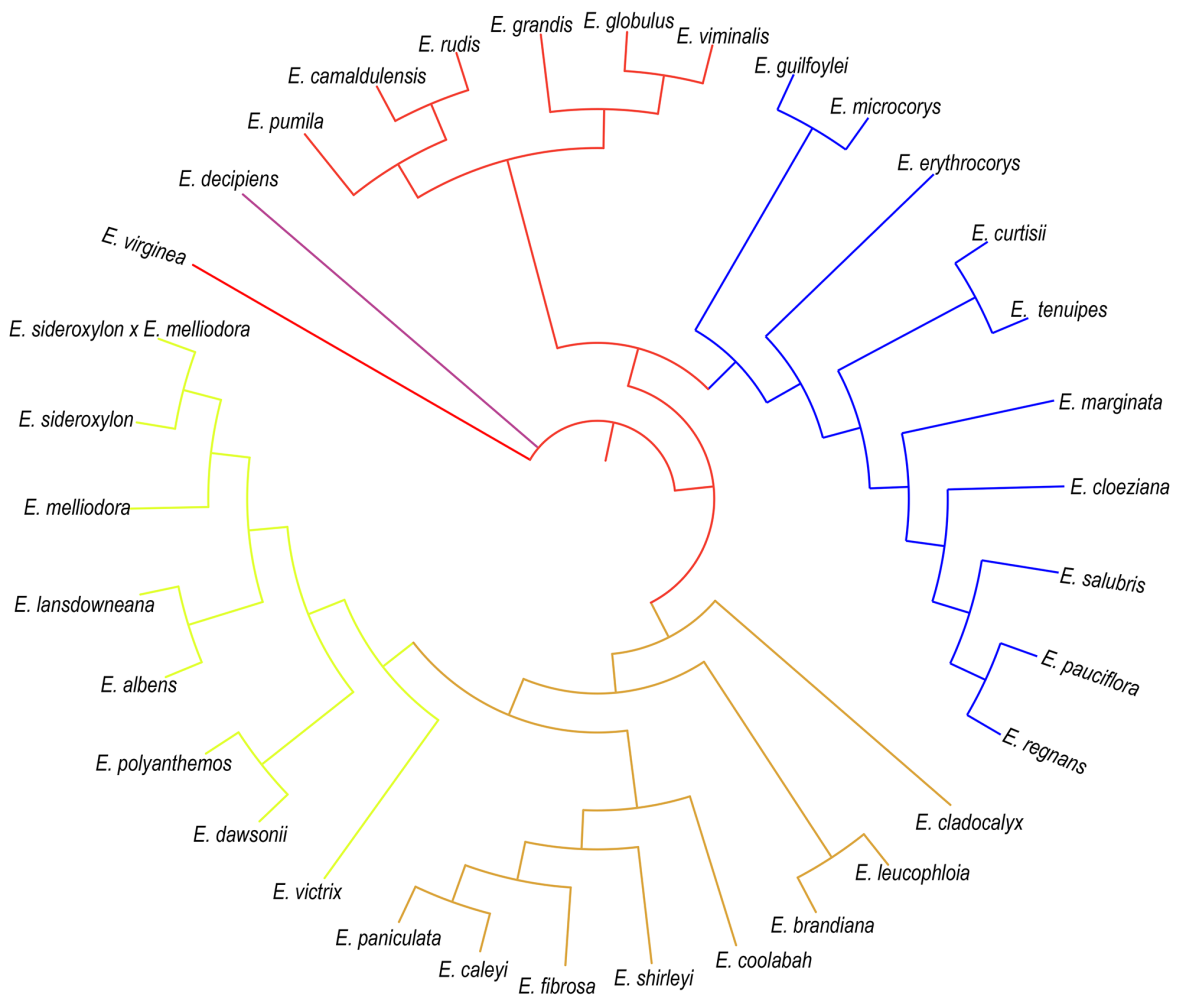
The SNP-based phylogeny of 34 Eucalyptus species

**Fig. 4:**
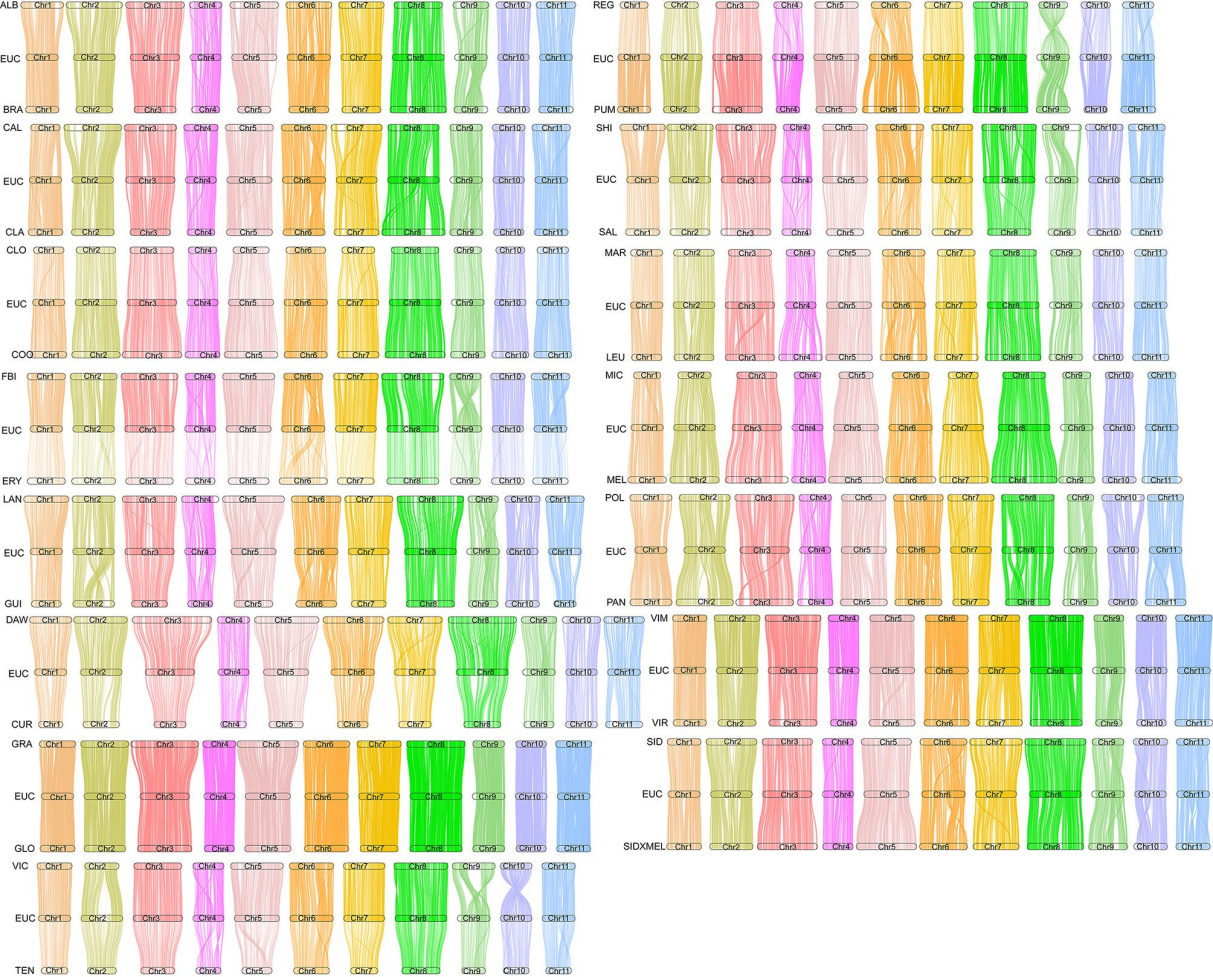
Comparative genomes resolve genome synteny in 31 eucalyptus species

To understand phylogenetic relationships, 34 Eucalyptus accessions were collected (Supplementary Table 10). A phylogenetic tree was constructed using 798,492 high-quality SNPs, which revealed that the tree was divided into six branches. The phylogenetic relationship between Eucalyptus species showed that *E. virginea* (VIR) and *E. decipiens* (DEC) were clearly separated (Fig. 3). *E. globulus* (GLO) and *E. viminalis* (VIM) were the closest relatives. *E. albens* (ALB) and *E. polyanthemos* (POL) belonged to the same evolutionary branch. *E. curtisi* (CUR) and *E. tenuipes* (TEN) were closely related. *E. regnans* (REG) and *E. pauciflora* (PAU) belonged to the same evolutionary clade. To explore their evolutionary relationships, genomic synteny analyses of *E. urophylla × E. grandis* (EUC) and the other 30 Eucalyptus species were performed, which exhibited high levels of genomic synteny (Fig. 4). Interestingly, the comparative genome structure of EUC, *E. grandis*, and *E. globulus* (GLO**)** showed higher collinearity, indicating no large-scale structural variation after divergence, which was consistent with their evolutionary relationships (Fig. 3 and Supplementary Fig. 8). Interestingly, we found that Chr9 showed large structural variations among EUC, *E. fibrosa* (FBI), *E. brandiana* (BRA), *E lansdowneana* (LAN), *E. regnans* (REG), *E. pumila* (PUM), *E. shirleyi* (SHI), *E. polyanthemos* (POL), and *E. victrix* (VIC**)** according to the synteny analysis shown in Fig. 4. Large structural variations in chromosomes Chr2, Chr4, and Chr6 were found between EUC and *E. erythrocorys* (ERY) (Fig. 4). EUC and *E. guilfoylei* (GUI) showed large chromosomal rearrangements in Chr2 and Chr6 (Fig. 4). Chr3 and Chr8 showed large inversions between EUC and *E. paniculata* (PAN) (Fig. 4).

## Discussion

### The high quality *E. urophylla × E. grandis* genome provided community resources for Eucalyptus genetic breeding research

Myrtaceae is the 8th largest flowering plant family and is mainly grown in the subtropics and tropics, with 5950 species in 132 genera [17]. Eucalyptus is the most planted hardwood species worldwide and a member of the Myrtaceae family, showing unique economic value as a global renewable energy resource [16]. However, studies on *E. urophylla × E. grandis* genomics and molecular levels are limited and almost nonexistent. Resolving the genomic resources of *E. urophylla × E. grandis* is, therefore, of great significance, because it can promote eucalyptus evolutionary studies and molecular breeding. With the development of long-read sequencing techniques, complex heterozygous genomes have been successfully assembled [18–20]. Here, we completed a high-quality *E. urophylla × E. grandis* reference genome, which is an economically valuable source of natural products and accelerates the application for molecular breeding, evolution, and genetics of Myrtaceae.

We generated a high-quality genome assembly of 545.75 Mb for *E. urophylla × E. grandis*, which was smaller than that of *E. grandis* (605 Mb) [16]. The genome scaffold N50 was 51.62 Mb, which is larger than that of other Myrtaceae family, such as *Psidium guajava* (443.8 Mb, N50 40.4 Mb) [21]. We predicted 34 and 502 protein-coding genes. This number was higher than that in *Psidium guajava* [21] and lower than that in *E. grandis* [16]. As an important component of genome composition, repetitive sequences play crucial roles in chromosomal rearrangement, gene regulation, and genome evolution, but also have significantly affect high-quality genome assembly. Here, we found 58.56% repeated sequences in the *E. urophylla × E. grandis* genome, with 39.4% LTRs, which was higher than those in *E. grandis* and *Psidium guajava*. Furthermore, BUSCO analysis revealed that 98.4% of the highly conserved core proteins supported the assembly integrity of genetic regions, indicating that the reference genome quality was superior to that of the guava genome (95.7%) [21]. Overall, the assembled *E. urophylla × E. grandis* genome in this study was complete and accurate, providing valuable genomic resources for subsequent studies on Eucalyptus population evolution and genetic improvement.

### Phylogenetic analysis contributed to the evolutionary relationship

To explore the phylogenetic analyses, 13 other genomes of representative plant species were selected, which showed *E. urophylla × E. grandis* were most closely related to *E. grandis*, supporting the placement of *E. urophylla × E. grandis* and *E. grandis* in Myrtaceae, and *P. granatum* in the order Myrtales. WGD events can cause gene family expansion, chromosomal rearrangement, genome size variation, and species evolution [22]. Gene family analysis was performed for *E. urophylla × E. grandis*, *E. grandis*, *Arabidopsis*, *M. domestica*, and *C. papaya*, which revealed 8882 common gene families. In contrast, the *E. urophylla × E. grandis* genome had unique 850 gene families, which was more than those of *E. grandis* and less than those of *M. domestica*, *Arabidopsis*, and *C. papaya*. The Ks analysis revealed that *E. urophylla × E. grandis* shared a recent WGD event with *E. grandis* and *P. granatum* (Fig. 2d). The chromosomal regions of *E. urophylla × E. grandis* showed a one-to-one correspondence with *E. grandis* (Fig. 4), which is consistent with an evolutionary relationship (Fig. 3), possibly because they did not have large-scale structural variation after species divergence (Fig. 4).

Because genome research on eucalyptus is still relatively lacking, there is some controversy regarding the classification of eucalyptus. To understand phylogenetic relationships, 34 Eucalyptus species accessions were collected (Supplementary Table 10). A phylogenetic tree was constructed using 798,492 high-quality SNPs, which showed that the phylogenetic tree was divided into six branches (Fig. 3). The phylogenetic relationship between eucalyptus species showed that VIR and DEC were clearly separated. GLO and VIM were the closest relatives. ALB and POL belonged to the same evolutionary branch. Similarly, CUR and TEN were closely related. REG and PAU belonged to the same evolutionary clade. These results are consistent with those of previous studies [23] and contribute to our understanding of the evolutionary relationships between different Eucalyptus species at the genome level.

### Comparative genomics reveals interspecific structural variation in Eucalyptus

Studying structural variations (SVs) is a challenging yet important for understanding trait differences in highly repetitive genomes as well as an important component of genetic diversity, and has important implications in evolution and breeding. However, owing to the relative lag in eucalyptus genomic research, eucalyptus research remains in its infancy. Genomic synteny analyses showed that our genome assembly of *E. urophylla × E. grandis* had a high level of genome synteny with other Eucalyptus species. Interestingly, the comparative genome structures of EUC, GRA, GLO, and *E. viminalis* (VIM) showed higher collinearity, indicating no large-scale structural variation after divergence, which was also consistent with their evolutionary relationships (Fig. 3 and Supplementary Fig. 8). Structural variation was weighed more heavily for genomic and trait effects. Interestingly, we found that chromosomes Chr2, Chr3, Chr6, Chr8, and Chr9 showed large structural variations, which may be an important reason underlying the differentiation of these eucalyptus species. This lays the foundation for our follow-up research on important characteristics such as material and stress resistance.

## Conclusions

Overall, in this study, we assembled 545.75 Mb of the *E. urophylla × E. grandis* high-quality genome and annotated 34,502 protein-coding genes, which demonstrated a complete genome landscape. Comparative genomic analysis revealed that *E. urophylla × E. grandis* underwent a recent WGD event. We characterized the phylogenetic relationships of 34 eucalypts at the genome-wide level for the first time. Interspecific structural variations were identified using genomic collinearity analysis. This will accelerate the application of molecular genetic breeding of *E. urophylla × E. grandis*, deepen our understanding of eucalyptus biology and genetic improvement of eucalyptus, and lay the foundation for population genome research.

## Materials and methods

### Library construction and sequencing of ***E. urophylla × E. grandis***

Young leaves of *E. urophylla × E. grandis* plants were collected and immediately frozen in liquid nitrogen. We extracted DNA from fresh young leaves for Illumina and PacBio sequencing with a QIAGEN® Genomic Kit (QIAGEN, Germany). Sequencing libraries for Illumina, PacBio HiFi, and Hi-C were constructed.

### Genome assembly and correction

K-mer analysis was used to estimate genomic size, heterozygosity, and repeats by plotting the 17-mer depth distribution [24, 25]. HiFi data were assembled with the Hifiasm software using default parameters [26]. Juicer (v.1.5) [27], a 3D-DNA scaffolding pipeline [28], Juicebox (v.1.11.08) [29], and HiCUP [30] were used to correct the initial orientations for genome assembly. BUSCO analyses were used to evaluate the completeness of the reference assembly (https://busco.ezlab.org/frame_wget.html) [31]. Genome assembly continuity was evaluated using the LTR assembly index (LAI) [32], as described by Ou et al. [29].

### Repetitive sequence and noncoding RNA annotation

Complementary methods were used to identify repetitive sequences as described by Shen et al. [4]. Tandem Repeats Finder (v4.09) software [33] was used to identify tandem repeats. TEs were predicted using a complementary strategy with RepeatMasker (v4.06), LTR_FINDER (v1.05) [34], RepeatProteinMasker, RepeatScout (v1.05) [35], and RepeatModeler (v1.05). Eventually, we obtained a non-redundant genome. The miRNAs, tRNA, rRNA, and snRNAs were annotated. The tRNAs were identified using tRNAscan-SE (v1.3.1) [36]. The rRNAs were predicted using BLAST [37]. The Rfam database (release 13.0) [38] was used to search for snRNAs and miRNAs using Infernal (v1.1) [39].

### Prediction and annotation of *E. urophylla × E. grandis*

Protein-coding gene prediction was performed using three independent methods as previously described [4]. Five programs were used to conduct *de novo* prediction: GlimmerHMM (v3.0.4) [40], Augustus (v3.2.1) [41], Genscan (v1.0) [42], GeneID (v1.4.4) [43], and SNAP [44]. Five representative species (*R. argentea*, *P. granatum* [45], *C. citriodora* [46], *S. oleosum*, and *E. grandis* [47]) were used for homolog-based predictions using the GeMoMa [48] software. Transcriptome assembly prediction was performed using the Hisat [49] and Stringtie [50]. Next, we integrated the gene sets into a non-redundant with the MAKER2 [51]. Functional annotations were performed using InterProScan [52], TrEMBL [53], NCBI-NR (V2013), SwissProt [53], Pfam (http://pfam.xfam.org), GO [54], KOG, and KEGG [55] (Table S3). We obtained a reliable set of gene annotations by integrating 1549 (96.00%) complete BUSCO results.

### Gene family identification and evolutionary analysis

We used OrthoMCL (v2.0.9) to obtain gene clusters from *E. urophylla × E. grandis* and 12 other species genomes obtained from the phytozome database (https://data.jgi.doe.gov/), including *A. thaliana* (TAIR10), *C. papaya* (ASGPBv0.4), *E. grandis* (v2.0), *M. domestica* (HFTH1), *O. sativa* (v7.0), *P. trichocarpa* (v4.1), *P. persica* (v2.1), *P. granatum* (GCF_007655135.1_ASM765513v2), *S. lycopersicum* (ITAG4.1), *T. cacao* (v2.1), *V. vinifera* (v2.1), and *Z. jujuba* (GCF_000826755.1_ZizJuj_1.1) with default parameters. Subsequently, we obtained gene families (Supplementary Table S9) and generated a shared gene family subset for five species (*E. urophylla × E. grandis*, *A. thaliana*, *M. domestica*, *E. grandis*, and *C. papaya*) (Fig. 2c). The divergence times was estimated to use the MCMCTree program (v4.9) [56] with the parameters “the clock = 3 and model = 0”. CAFÉ (v4.2) [57] was used to identify gene family expansion and contraction. The results of gene family expansions or contractions were obtained as described by Chen et al. [20]. WGD analysis was performed using BLASTP (E-value = 1 × 10^− 10^), ML estimation in CODEML (v4.9) of the PAML software [56], and Multi-tAxon Paleopolyploidy Search software [58] based on age distributions [20, 59] for the three selected Myrtales species: *E. grandis*, *P. granatum*, and *E. urophylla × E. grandis*. Finally, the WGD ages described by Chen et al. [20] were estimated. We first used MUMMER [60] to call SNPs, then used a script to convert the output format of the MUMMER software to VCF, and used bcftools (http://github.com/samtools/bcftools) to merge. A neighbor-joining phylogeny was constructed using VCF2Dis (https://github.com/BGI-shenzhen/VCF2Dis) based on the P-distance matrix. Finally, the matrix file was uploaded to a website (http://www.atgc-montpellier.fr/fastme/) to obtain the tree file. Comparative genomic analyses were performed using MUMmer (V4.0) [60] as described by Shen et al. [4].

## Electronic supplementary material

Below is the link to the electronic supplementary material.

Supplementary Material 1

Supplementary Material 2

Supplementary Material 3

Supplementary Material 4

Supplementary Material 5

Supplementary Material 6

Supplementary Material 7

Supplementary Material 8

Supplementary Material 9

Supplementary Material 10

Supplementary Material 11

## Data Availability

Raw sequencing data were deposited in the National Genomics Data Center under accession numbers PRJCA012131 and CRA008320 (https://ngdc.cncb.ac.cn/gsa/s/8G10iTuf). Genomic data were obtained from https://figshare.com/s/c01f66c30a7296134936 [61].

## References

[1] Ouyang L, Wang Z, Li L, Chen B (2020). Physiological parameters and differential expression analysis of N -phenyl- N′ -[6-(2-chlorobenzothiazol)-yl] urea-induced callus of *Eucalyptus urophylla* × *Eucalyptus grandis*. PeerJ.

[2] Chen S, Zheng J, Liu X. Hundred year histories and prospect of *eucalyptus* cultivation technology development in China. World For Res. 2018.

[3] Wang M, Tu L, Yuan D, Zhu D, Shen C, Li J (2019). Reference genome sequences of two cultivated allotetraploid cottons, *Gossypium hirsutum* and *Gossypium barbadense*. Nat Genet.

[4] Shen C, Wang N, Zhu D, Wang P, Wang M, Wen T (2021). *Gossypium tomentosum* genome and interspecific ultra-dense genetic maps reveal genomic structures, recombination landscape and flowering depression in cotton. Genomics.

[5] Jaillon O, Aury JM, Noel B, Policriti A, Clepet C, Casagrande A (2007). The grapevine genome sequence suggests ancestral hexaploidization in major angiosperm phyla. Nature.

[6] Tang H, Bowers JE, Wang X, Ming R, Alam M, Paterson AH (2008). Synteny and collinearity in plant genomes. Science.

[7] Li J, Wang Y, Dong Y, Zhang W, Wang D, Bai H (2021). The chromosome-based lavender genome provides new insights into Lamiaceae evolution and terpenoid biosynthesis. Hortic Res.

[8] Kang M, Wu H, Yang Q, Huang L, Hu Q, Ma T (2020). A chromosome-scale genome assembly of Isatis indigotica, an important medicinal plant used in traditional chinese medicine: an Isatis genome. Hortic Res.

[9] Wei S, Yang Y, Yin T (2020). The chromosome-scale assembly of the willow genome provides insight into *Salicaceae* genome evolution. Hortic Res.

[10] Chen SP, Sun WH, Xiong YF, Jiang YT, Liu XD, Liao XY (2020). The *Phoebe* genome sheds light on the evolution of magnoliids. Hortic Res.

[11] Qin X, Zhang Z, Lou Q, Xia L, Li J, Li M (2021). Chromosome-scale genome assembly of Cucumis hystrix-a wild species interspecifically cross-compatible with cultivated cucumber. Hortic Res.

[12] Brooker MIH (2000). A new classification of the genus *Eucalyptus* (Myrtaceae). Aust Syst Bot.

[13] Slee A, Brooker M, Duffy S, West J (2006). EUCLID: eucalypts of Australia.

[14] Grattapaglia D, Vaillancourt RE, Shepherd M, Thumma BR, Foley W, Külheim C (2012). Progress in Myrtaceae genetics and genomics: *eucalyptus* as the pivotal genus. Tree Genet Genomes.

[15] Butler JB, Vaillancourt RE, Potts BM, Lee DJ, King GJ, Baten A (2017). Comparative genomics of *Eucalyptus* and Corymbia reveals low rates of genome structural rearrangement. BMC Genomics.

[16] Myburg AA, Grattapaglia D, Tuskan GA, Hellsten U, Hayes RD, Grimwood J (2014). The genome of *Eucalyptus grandis*. Nature.

[17] Christenhusz MJM, Byng JW (2016). The number of known plants species in the world and its annual increase. Phytotaxa.

[18] Wenger AM, Peluso P, Rowell WJ, Chang PC, Hall RJ, Concepcion GT (2019). Accurate circular consensus long-read sequencing improves variant detection and assembly of a human genome. Nat Biotechnol.

[19] Hon T, Mars K, Young G, Tsai YC, Karalius JW, Landolin JM (2020). Highly accurate long-read HiFi sequencing data for five complex genomes. Sci Data.

[20] Chen F, Su L, Hu S, Xue JY, Liu H, Liu G (2021). A chromosome-level genome assembly of rugged rose (*Rosa rugosa*) provides insights into its evolution, ecology, and floral characteristics. Hortic Res.

[21] Feng C, Feng C, Lin X, Liu S, Li Y, Kang M (2021). A chromosome-level genome assembly provides insights into ascorbic acid accumulation and fruit softening in guava (*Psidium guajava*). Plant Biotechnol J.

[22] El Baidouri M, Panaud O (2013). Comparative genomic paleontology across plant kingdom reveals the dynamics of TE-driven genome evolution. Genome Biol Evol.

[23] Woodhams M, Steane DA, Jones RC, Nicolle D, Moulton V, Holland BR (2013). Novel distances for dollo data. Syst Biol.

[24] Kajitani R, Toshimoto K, Noguchi H, Toyoda A, Ogura Y, Okuno M (2014). Efficient de novo assembly of highly heterozygous genomes from whole-genome shotgun short reads. Genome Res.

[25] Li R, Zhu H, Ruan J, Qian W, Fang X, Shi Z (2010). De novo assembly of human genomes with massively parallel short read sequencing. Genome Res.

[26] Cheng H, Concepcion GT, Feng X, Zhang H, Li H (2021). Haplotype-resolved de novo assembly using phased assembly graphs with hifiasm. Nat Methods.

[27] Durand NC, Shamim MS, Machol I, Rao SS, Huntley MH, Lander ES (2016). Juicer provides a one-click system for analyzing loop-resolution Hi-C experiments. Cell Syst.

[28] Dudchenko O, Batra SS, Omer AD, Nyquist SK, Hoeger M, Durand NC (2017). De novo assembly of the Aedes aegypti genome using Hi-C yields chromosome-length scaffolds. Science.

[29] Durand NC, Robinson JT, Shamim MS, Machol I, Mesirov JP, Lander ES (2016). Juicebox provides a visualization system for Hi-C contact maps with unlimited zoom. Cell Syst.

[30] Wingett S, Ewels P, Furlan-Magaril M, Nagano T, Schoenfelder S, Fraser P (2015). HiCUP: pipeline for mapping and processing Hi-C data. F1000Res.

[31] Simão FA, Waterhouse RM, Ioannidis P, Kriventseva EV, Zdobnov EM (2015). BUSCO: assessing genome assembly and annotation completeness with single-copy orthologs. Bioinformatics.

[32] Ou S, Chen J, Jiang N (2018). Assessing genome assembly quality using the LTR Assembly Index (LAI). Nucleic Acids Res.

[33] Benson G (1999). Tandem repeats finder: a program to analyze DNA sequences. Nucleic Acids Res.

[34] Xu Z, Wang H (2007). LTR_FINDER: an efficient tool for the prediction of full-length LTR retrotransposons. Nucleic Acids Res.

[35] Price AL, Jones NC, Pevzner PA (2005). De novo identification of repeat families in large genomes. Bioinformatics.

[36] Lowe TM, Eddy SR (1997). TRNAscan-SE: a program for improved detection of transfer RNA genes in genomic sequence. Nucleic Acids Res.

[37] Kent WJ (2002). BLAT–the BLAST-like alignment tool. Genome Res.

[38] Griffiths-Jones S, Moxon S, Marshall M, Khanna A, Eddy SR, Bateman A (2005). Rfam: annotating non-coding RNAs in complete genomes. Nucleic Acids Res.

[39] Nawrocki EP, Eddy SR (2013). Infernal 1.1: 100-fold faster RNA homology searches. Bioinformatics.

[40] Majoros WH, Pertea M, Salzberg SL (2004). TigrScan and GlimmerHMM: two open source ab initio eukaryotic gene-finders. Bioinformatics.

[41] Stanke M, Waack S (2003). Gene prediction with a hidden Markov model and a new intron submodel. Bioinformatics.

[42] Burge C, Karlin S (1997). Prediction of complete gene structures in human genomic DNA. J Mol Biol.

[43] Blanco E, Parra G, Guigó R. Using geneid to identify genes. Curr Protoc Bioinformatics. 2007;Chapter:Unit 4.3. 10.1002/0471250953.bi0403s18 18428791

[44] Korf I (2004). Gene finding in novel genomes. BMC Bioinformatics.

[45] Qin G, Xu C, Ming R, Tang H, Guyot R, Kramer EM (2017). The pomegranate (*Punica granatum* L.) genome and the genomics of punicalagin biosynthesis. Plant J.

[46] Healey AL, Shepherd M, King GJ, Butler JB, Freeman JS, Lee DJ (2021). Pests, diseases, and aridity have shaped the genome of *Corymbia citriodora*. Commun Biol.

[47] Bartholomé J, Mandrou E, Mabiala A, Jenkins J, Nabihoudine I, Klopp C (2015). High-resolution genetic maps of Eucalyptus improve Eucalyptus grandis genome assembly. New Phytol.

[48] Keilwagen J, Wenk M, Erickson JL, Schattat MH, Grau J, Hartung F (2016). Using intron position conservation for homology-based gene prediction. Nucleic Acids Res.

[49] Kim D, Langmead B, Salzberg SL (2015). HISAT: a fast spliced aligner with low memory requirements. Nat Methods.

[50] Pertea M, Pertea GM, Antonescu CM, Chang TC, Mendell JT, Salzberg SL (2015). StringTie enables improved reconstruction of a transcriptome from RNA-seq reads. Nat Biotechnol.

[51] Holt C, Yandell M (2011). MAKER2: an annotation pipeline and genome-database management tool for second-generation genome projects. BMC Bioinformatics.

[52] Jones P, Binns D, Chang HY, Fraser M, Li W, McAnulla C (2014). InterProScan 5: genome-scale protein function classification. Bioinformatics.

[53] Bairoch A, Apweiler R (2000). The SWISS-PROT protein sequence database and its supplement TrEMBL in 2000. Nucleic Acids Res.

[54] Ashburner M, Ball CA, Blake JA, Botstein D, Butler H, Cherry JM (2000). Gene ontology: tool for the unification of biology. The Gene Ontology Consortium. Nat Genet.

[55] Kanehisa M, Goto S (2000). KEGG: kyoto encyclopedia of genes and genomes. Nucleic Acids Res.

[56] Paml YZ (2007). Phylogenetic analysis by maximum likelihood. Mol Biol Evol.

[57] De Bie T, Cristianini N, Demuth JP, Hahn MW (2006). CAFE: a computational tool for the study of gene family evolution. Bioinformatics.

[58] Li Z, Baniaga AE, Sessa EB, Scascitelli M, Graham SW, Rieseberg LH (2015). Early genome duplications in conifers and other seed plants. Sci Adv.

[59] Wang D, Zhang Y, Zhang Z, Zhu J, Yu J (2010). KaKs_Calculator 2.0: a toolkit incorporating gamma-series methods and sliding window strategies. Genomics Proteom Bioinf.

[60] Delcher AL, Phillippy A, Carlton J, Salzberg SL (2002). Fast algorithms for large-scale genome alignment and comparison. Nucleic Acids Res.

[61] Shen C, Li L, Ouyang L, Su M, Guo K. E. urophylla × E. grandis high quality genome and comparative genomics provide insight into evolution and diversification of Eucalyptus [Internet]. Figshare, 2023 [cited 2023 Jan 24]. 10.1186/s12864-023-09318-0 PMC10148406 37118687

