# Supplementary material for: E. urophylla × E. grandis high-quality genome and comparative genomics provide insights on evolution and diversification of eucalyptus
Source: BMC Genomics. 2023 Apr 28;24:223. doi: 10.1186/s12864-023-09318-0 (PMC10148406; doi:10.1186/s12864-023-09318-0)
Supplement: Supplementary file 11 — Supplementary Material 11 [file 12864_2023_9318_MOESM11_ESM.docx]

**
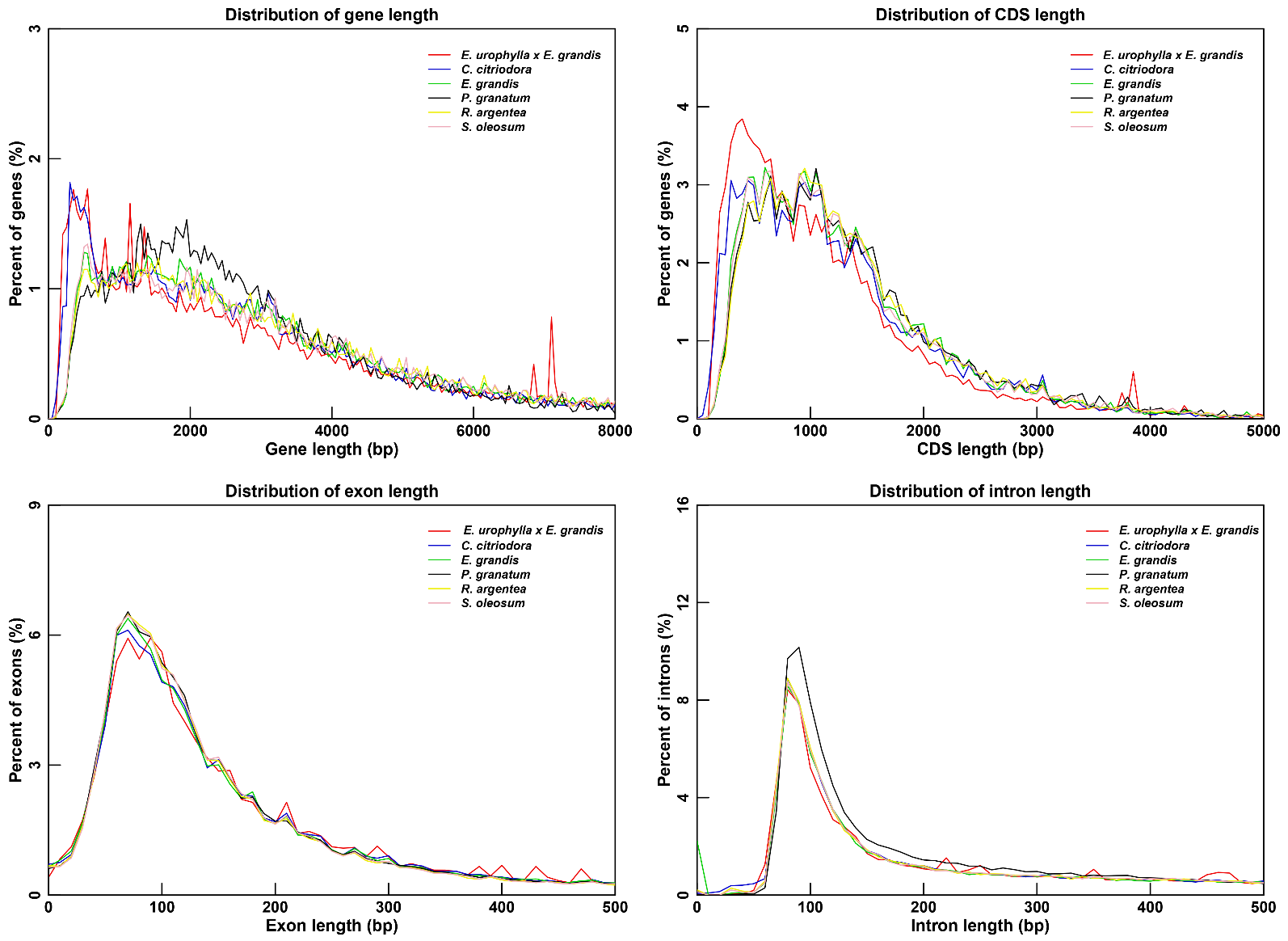
Supporting information**

Supplement Fig. 1 The gene structure prediction results.

**
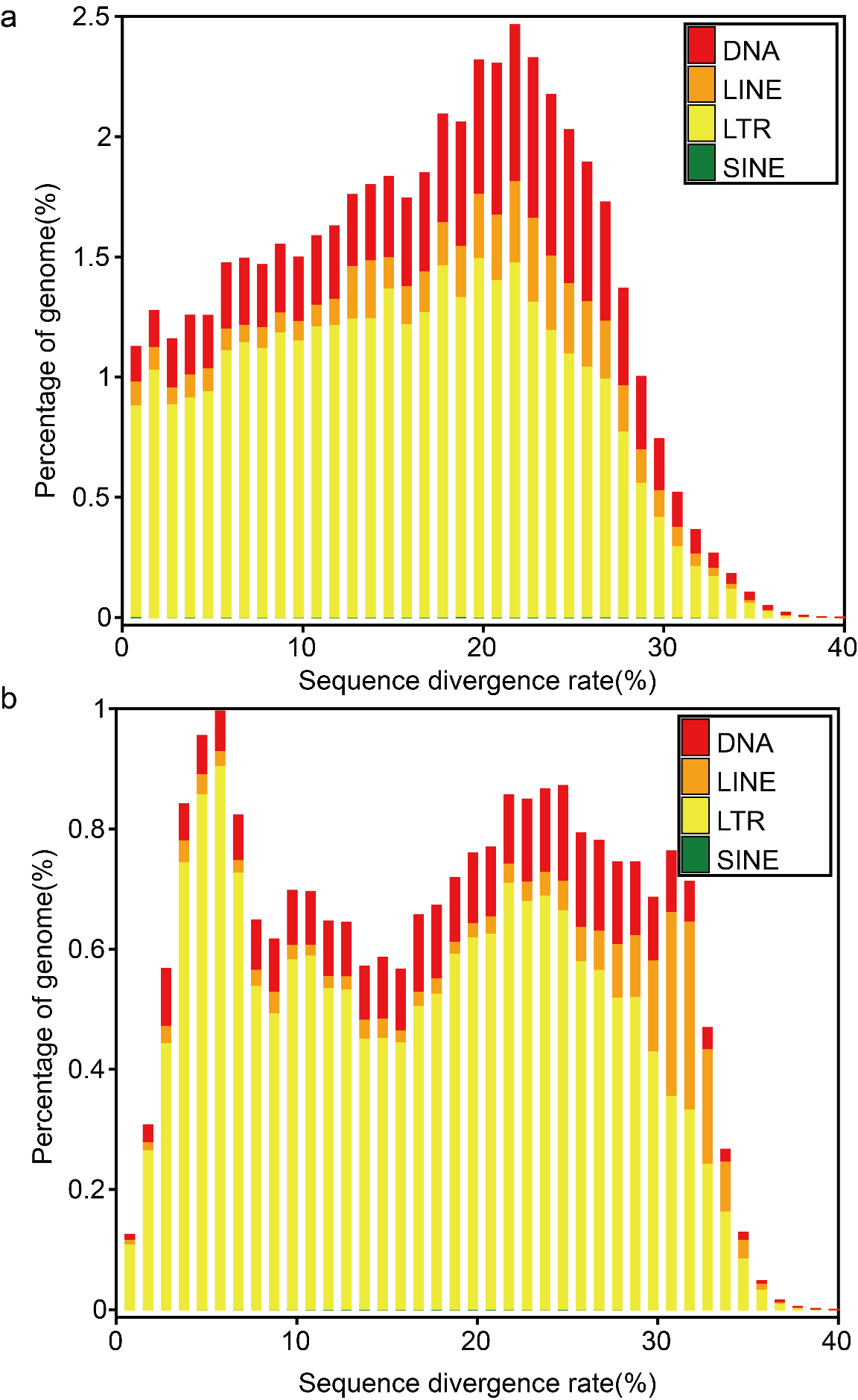
**

Supplement Fig. 2 Divergence distributions of four TE sequences predicted by the *De Novo* (a) and RepeatMasker (b).


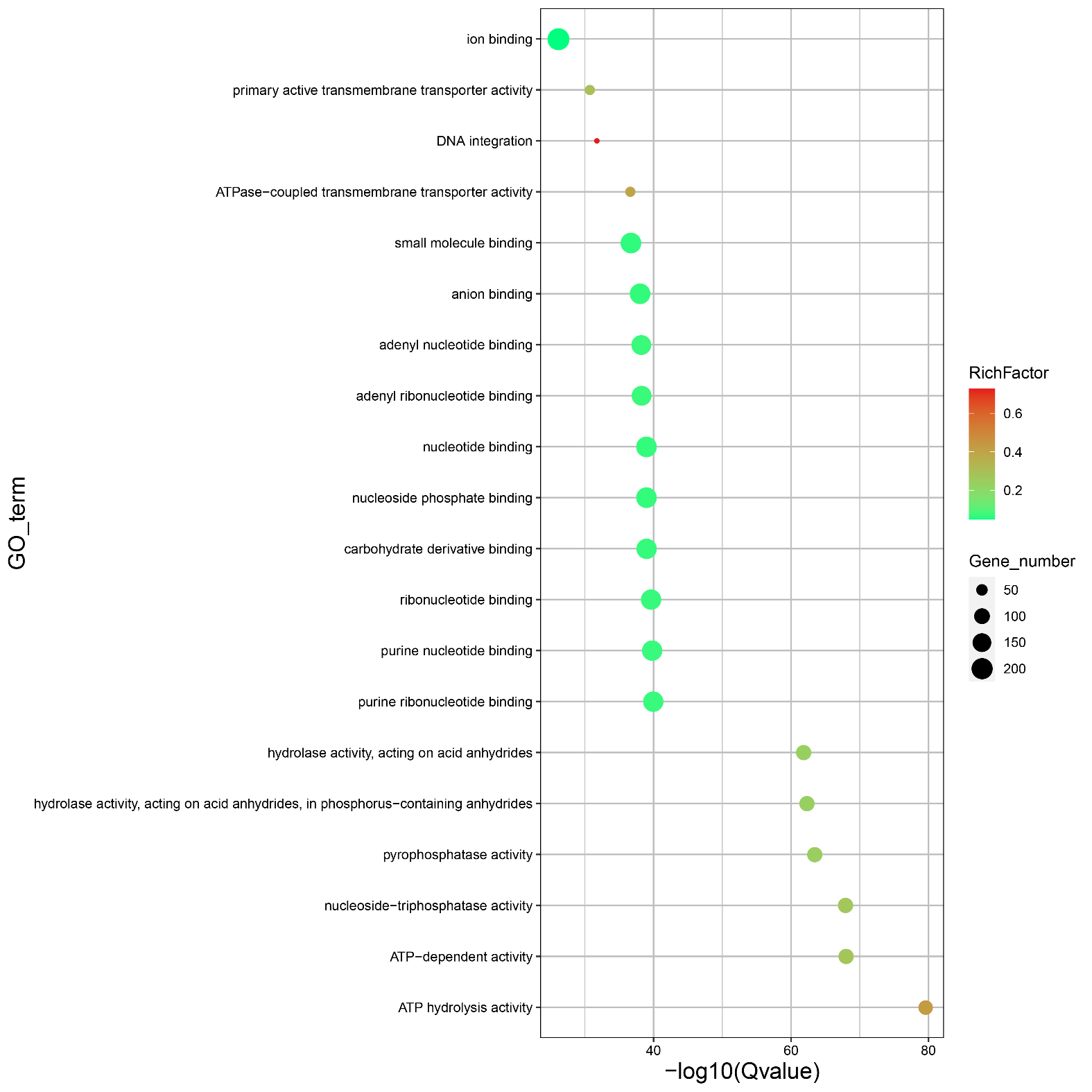
Supplement Fig. 3 GO enrichment results of genes in significantly expanded gene families.


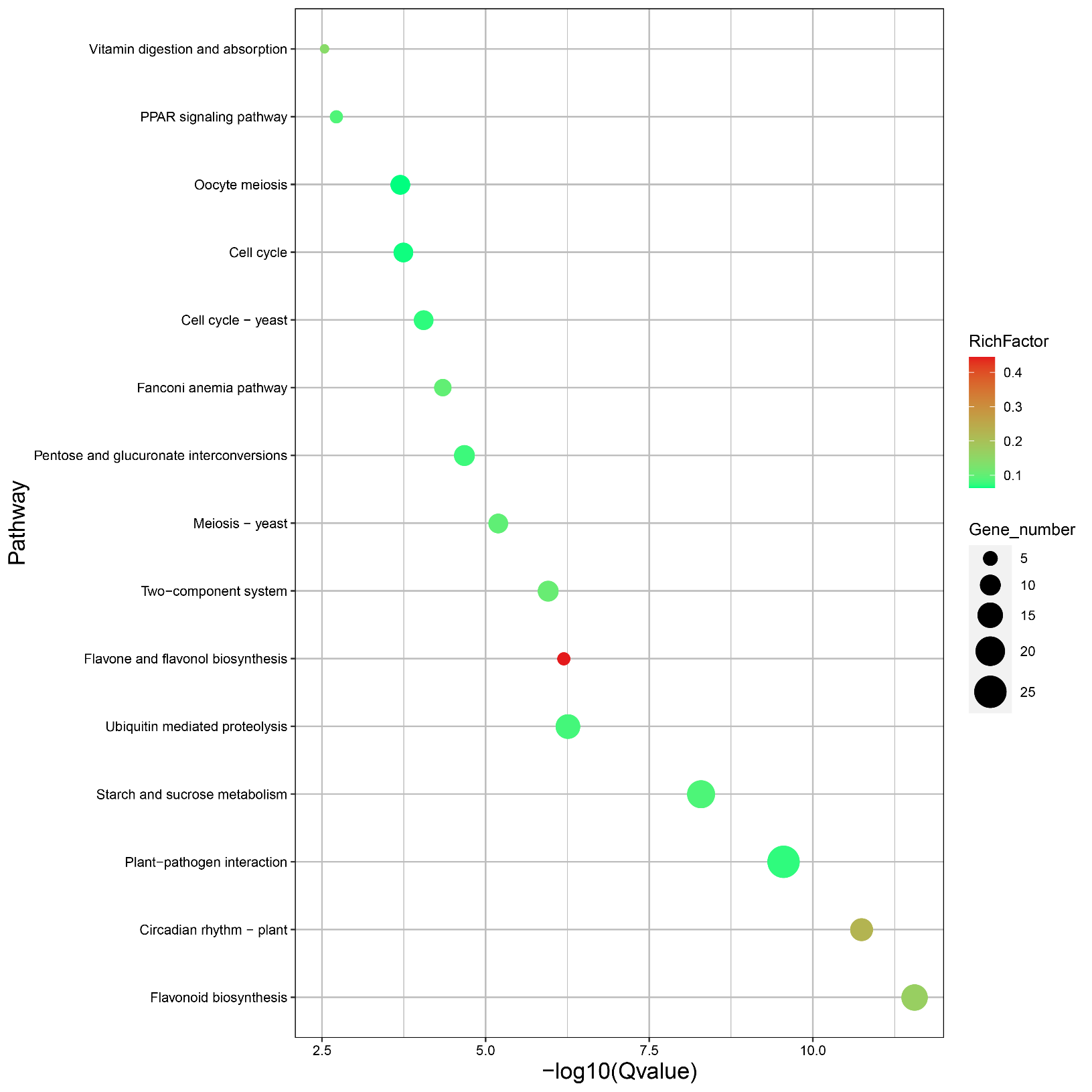
Supplement Fig. 4 KEGG enrichment results of genes in significantly expanded gene families.


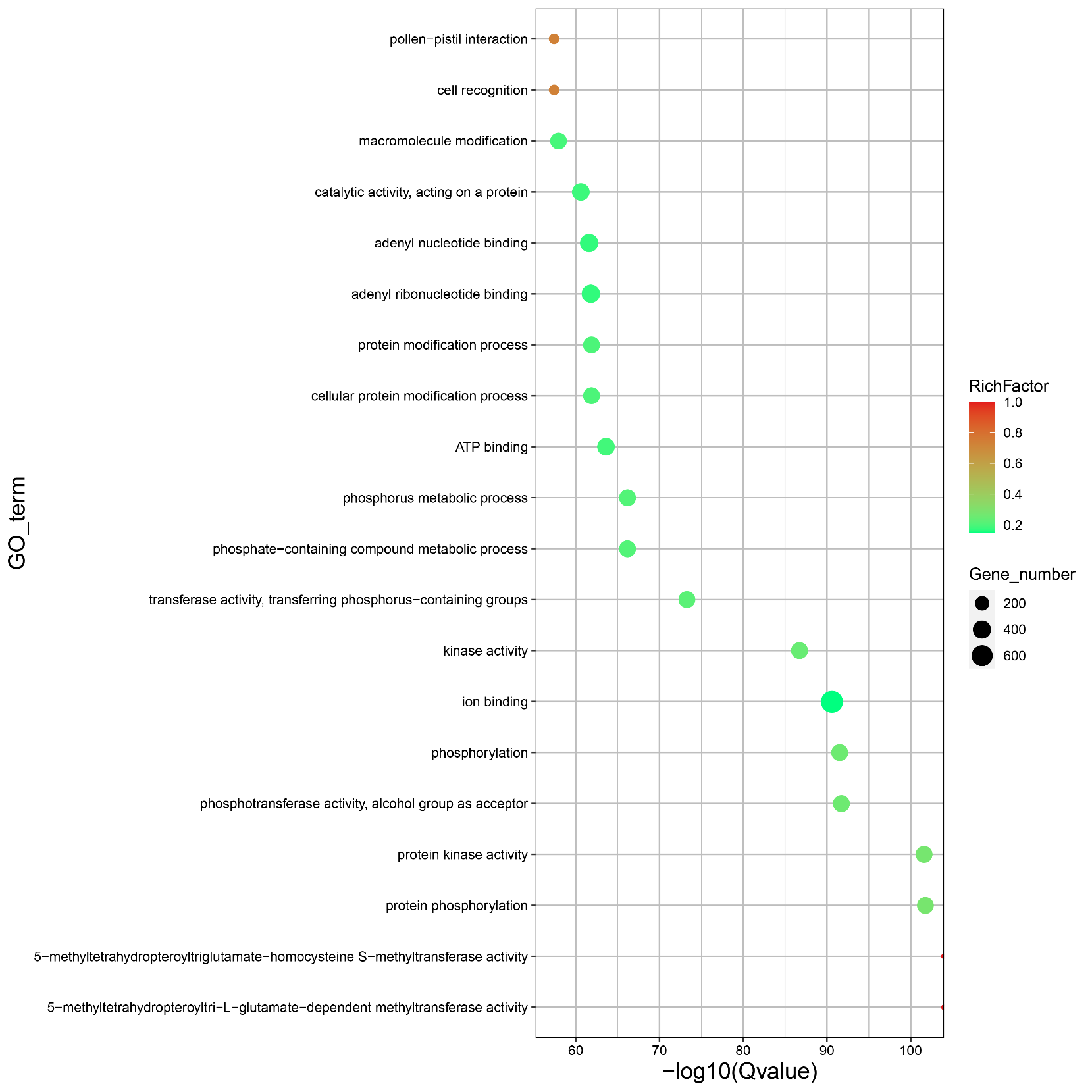
Supplement Fig. 5 GO enrichment results of genes in significantly constricted gene families.


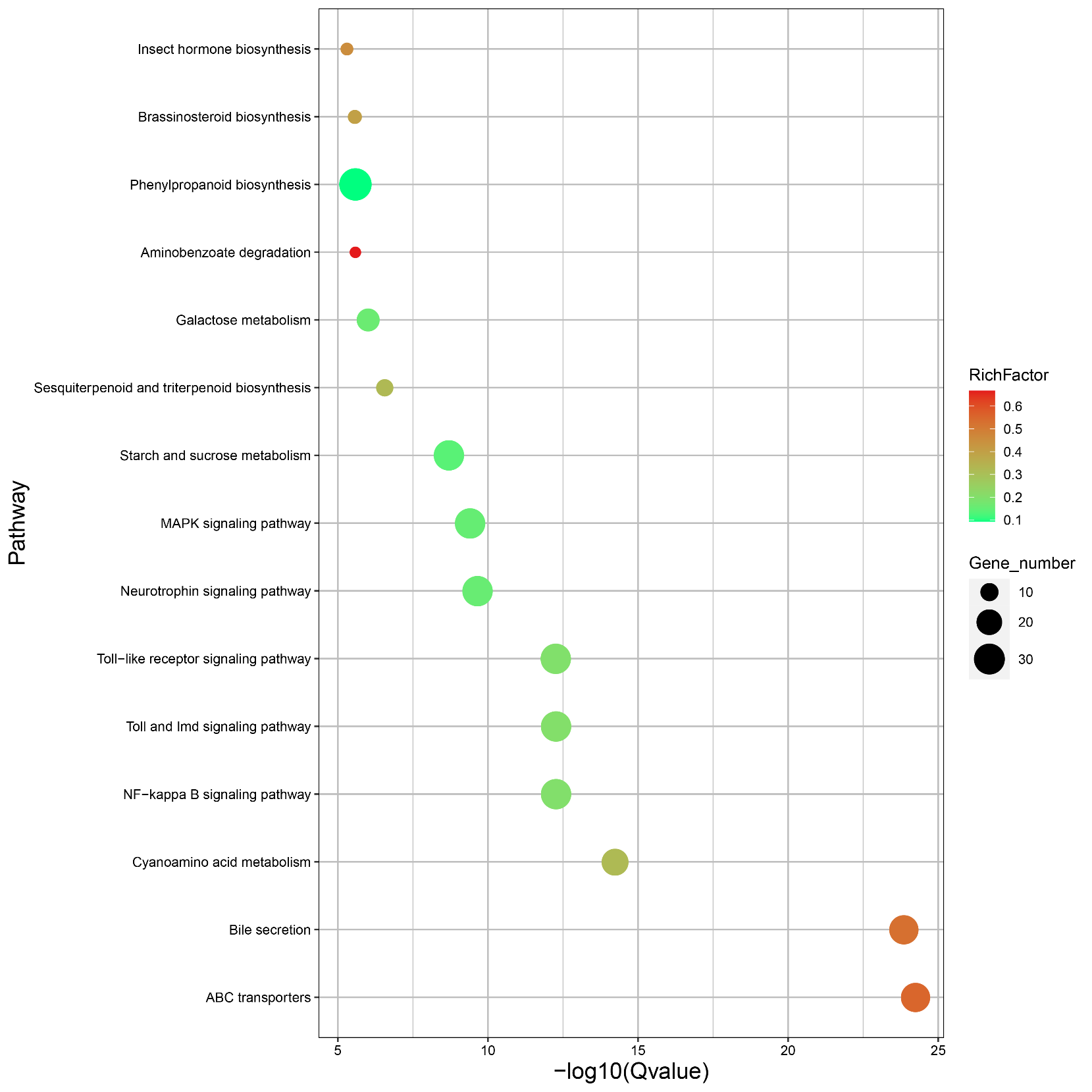
Supplement Fig. 6 KEGG enrichment results of genes in significantly constricted gene families.


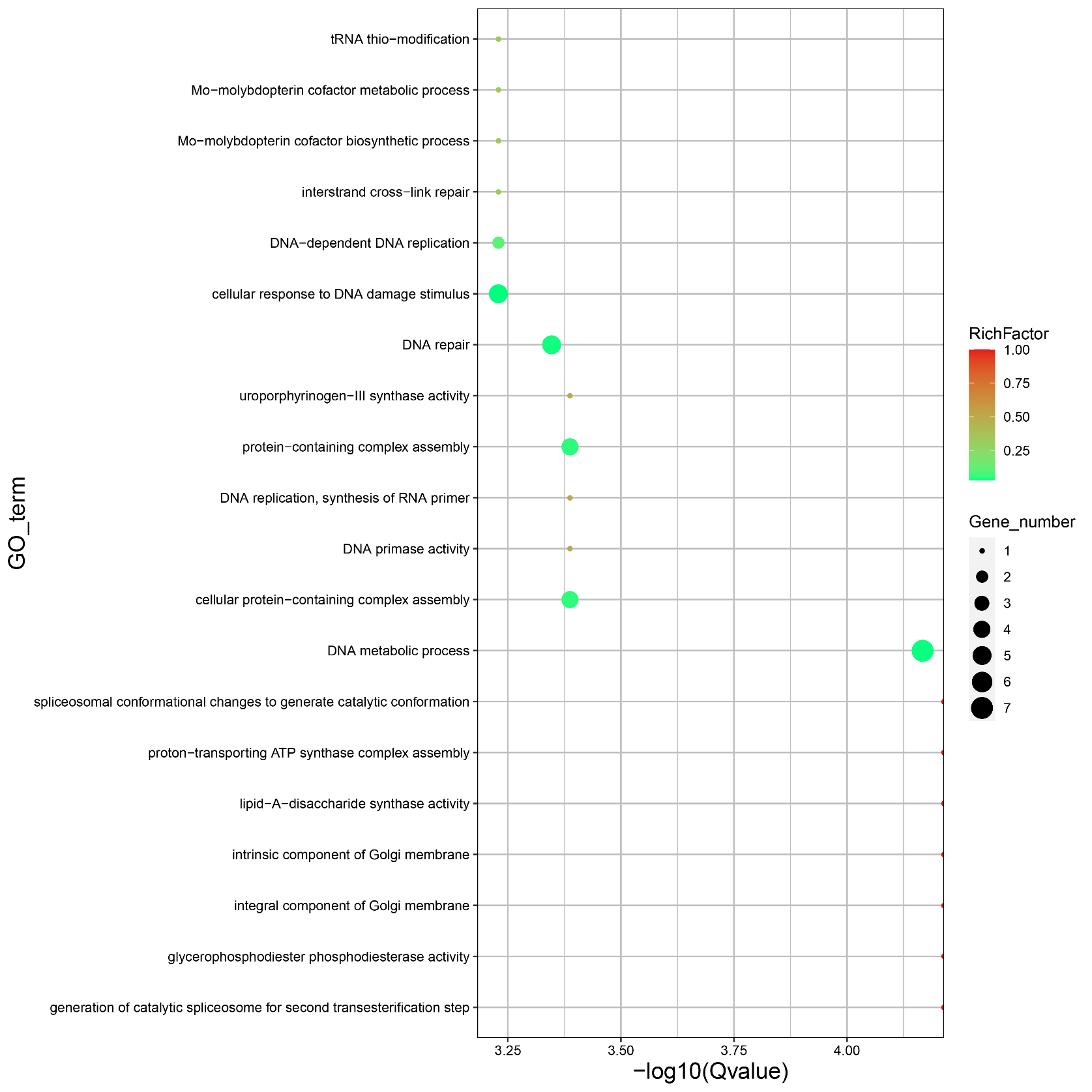
Supplement Fig. 7 GO enrichment results of positively selected genes.


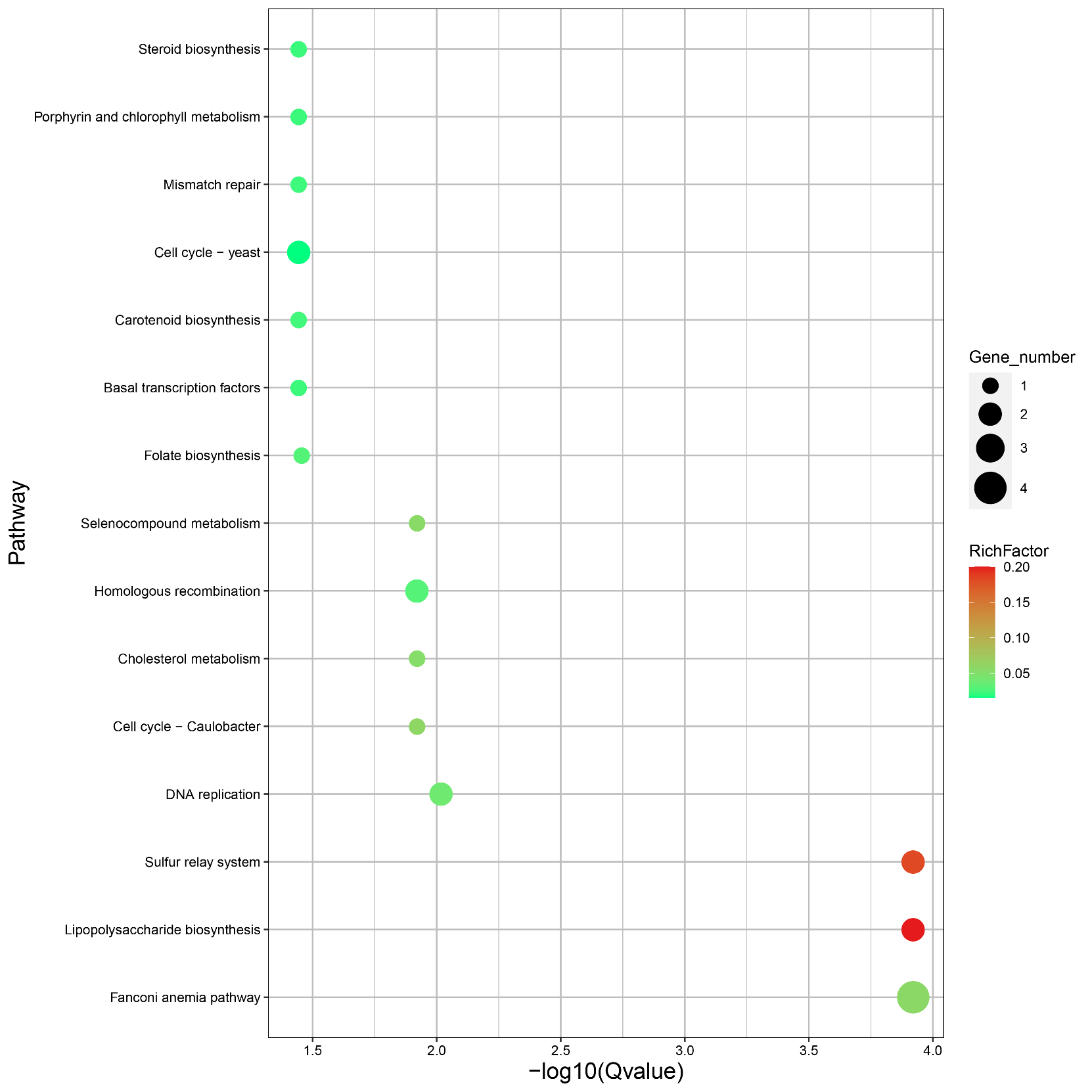
Supplement Fig. 8 KEGG enrichment results of positively selected genes.
